# Hospital and Institutional News

**Published:** 1912-07-20

**Authors:** 


### HOSPITAL AND INSTITUTIONAL NEWS.

### THE KING AND THE LEAGUE OF MERCY.

Last Saturday the King and Queen attended the garden party on behalf of the League of Mercy, which Mr. and Mrs. Leopold de Rothschild gave at Gunnersbury Park, Acton. Over 2,000 members of the League and their friends attended, and the terrace and the lawn sloping to the lake made a very pretty spectacle. Prince and Princess Alexander of Teck arrived at 4.30, and the King and Queen at 5 o'clock. The afternoon had a very pleasant air of informality about it, which the beautiful weather made most enjoyable. Their Majesties and Princess Mary strolled through the grounds, and, at some point or other, every member of the League had a ready opportunity of expressing his respect without incommoding the Royal visitors. The King and Queen, with a few privileged guests, then took tea in the dining-room, and left after a visit of almost two hours. The company included many distinguished people, not the least those whose work for the League of Mercy entitled them to wear its coveted decoration. It is not too much to say that the rank and file of the League, equally with its presidents and lady presidents, had on Saturday a privileged opportunity of expressing their gratitude to their Patron, Grand President and Lady Grand President, and all left sincerely grateful for the added pleasure which Mr. and Mrs. Leopold de Rothschild's hospitality had afforded them.

### A NEW WORKHOUSE INFIRMARY.

It was a happy thought that led the authorities at Haslingden in Lancashire to invite Mr. John Burns, the President of the Local Government Board, to open the new workhouse infirmary, and the ceremony lost nothing by its postponement to last Saturday to meet the convenience of a Minister, who, though well known, does not fill the newspapers so prominently as some of his younger confrères with more need or more wish for advertisement. The new infirmary is sufficiently large to accommodate 164 beds, and includes pavilions for men and women, a maternity ward, and a nurses' home, whilst there is additional space for the erection of two more pavilions if necessary. The principal wards are 24 feet wide, and are separated into compartments to permit of the classification of patients, and on each floor there are ward kitchens, store-rooms, and separation wards for isolation purposes, and lock wards are also provided. The total cost exceeds £30,000. The Union comprises the townships of Accrington, Bacup, Haslingden, and Rawtenstall, having a population of 117,000, an area of 27,000 acres, and a rateable value of £450,000. Mr. Burns, as was only to be expected, spoke mainly upon Poor-Law questions, and one statement he made was decidedly interesting. Although he had no wish to say an unkind word concerning the cost of infirmaries and hospitals, he regarded these merely as palliatives which were more costly than the remedies of a preventive character. That related, of course, to the poverty problem, but, we fear, a long long time will elapse before workhouse infirmaries can be dispensed with.

### PUBLIC HONOURS FOR A SCOTCH SUPERINTENDENT.

The final result of the controversy between Dr. William Butchart, till lately Medical Superintendent of the Blawarthill Fever Hospital, and the Renfrew and Clydebank Joint Hospital Board has now taken place by the public presentation to him of a silver casket containing an illuminated address and a purse of sovereigns from the inhabitants of Clydebank. As The Hospital recorded on January 27 last, Dr. Butchart was dismissed from his post because he refused at the dictation of the Renfrew and Clydebank Joint Hospital Board to adopt exclusively the Milne treatment. As we pointed out, he was fighting for the prerogative of medical superintendents all over the country, and on April 27 we had the satisfaction of reporting a good authority, who said that "for hospital cases at any rate there is nothing to be gained by adopting the strict Milne treatment." The presentation to Dr. Butchart on July 5, described on another page, has therefore an historic air.

### THE CULT OF THE SUN-BATH.

Medical opinion in this country has not perhaps encouraged so widely as in Germany the cult of the sun-bath, which now almost assumes the proportions of a fashionable craze. Our own climatic conditions place the regular sun-bather at a disadvantage as compared with the season ticket-holder at a Turkish bath establishment, but the impetus given last summer to helio-therapeutics has recruited enormously the ranks of the solarists. In Berlin the popularity of the sun-bath is extraordinary, and in the best districts of the city the most modern houses possess roof-gardens and erections which are to all intents and purposes lineal descendants of the Roman Solariums. In such delightful wooded resorts as the Grünewald and Wannsee, and also around the lakes between Potsdam and Berlin, may be seen large numbers of sun-bathers both in single spies and in club battalions. In England, since the days of the celebrated and isolated case of the lady on the Park Lane balcony, not a great deal has been heard of the therapeutic value of the sun-bath. But taken seriously and thoroughly there is no question that a moderate degree of insolation is well worth trying as an adjunct to the hygienic side of a holiday.

### THE DOCTORS AND SANATORIA BENEFIT.

The passage in the Chancellor of the Exchequer's speech last Saturday, which has perhaps excited most feeling, was that in connection with medical benefit, in which he said: "Then they say, 'You cannot supply the medical benefit because you cannot get the doctors.' Here is a very significant fact. Yesterday we had to find 11 tuberculosis doctors for the whole of Wales. . . . They got 57 applications, and the 11 were appointed yesterday, and they were mainly Englishmen." A significant comment on this is the announcement that two of the medical men appointed in Wales have intimated their intention of resigning the appointments.

Their names are Dr. D. A. Powell, of Charing Cross Hospital, and Dr. Cuthbert G. Welch, and the British Medical Association has recommended the other nine medical men to follow their example. In the meantime Sir William Plender's report on the earnings of doctors is exciting considerable criticism.

### PENNY FUNDS AND "POUND DAYS."

The supporters of the Worcestershire Hospital Penny Monthly Collection have been entertained at a garden party by Sir George and Miss Hingley at High Park, near Droitwich. Afterwards a meeting was held on the lawn, at which Mr. Francis Wood presided. He thanked all the workers for their continued help in collecting the monthly payments, which had resulted in a total of £344, which was a slight decrease (£33) on the previous year. He said that the result of the Insurance Act and of the examination of school children would be to increase the work of hospitals. The same afternoon a "Pound Day" was held in the infirmary, and from noon to 6 p.m. a steady stream of parcels poured into the Board Room. The term "pound" was interpreted with a generosity which would rejoice the heart of any housewife. Villages had combined and sent in hampers from outlying districts, while the children from some of the elementary schools came with their presents, and then visited the wards, which were thrown open to all visitors for the afternoon.

### THE REMOVAL OF HOPELESS CASES.

At the recent quarterly meeting of the Tiverton Joint Hospital Board, Dr. Liesching raised the question of the advisability of admitting "hopeless cases." "Do we," he asked "recruit the chances of those cases for recovery in removing them from their homes in a dying condition?" He urged that in very bad cases the written opinion of the patient's medical man should be obtained stating that it was to the patient's advantage to be admitted to a hospital. Mr. W. Dester, the chairman, pointed out that the risk to those in the patient's house should have at least equal consideration. Even though, as the Mayor, Mr. A. T. Gregory, suggested, the doctor is not called in early enough in many cases, and thus patients are often moribund when the order for their removal arrives, we agree with Mr. Dester, because there is sometimes a tendency in isolation hospitals to ignore everything else except the chance of a low death-rate. This tendency must be sternly repressed, and the danger to others in an infected house be placed second to no other interest. Indeed, no one at the meeting had sufficient logic to ask Dr. Liesching how, if a patient be moribund, his chances of recovery can be "recruited" by any action regarding him at all? We do not think a case was made out for their non-admission, nor that the additional process of a written authority from a medical man before removal is necessarily the best way to serve the true interests of the patients or the hospital.

### CARDIFF MEDICAL SCHOOL.

At a meeting of the Council of the University College of South Wales and Monmouthshire at Cardiff last week the announcement was formally conveyed in a letter from Colonel Bruce Vaughan of a gift of 10,000 guineas to defray the cost of a proposed new physiological block in connection with the Cardiff Medical School. It will be remembered that this gift was referred to in the issue of The Hospital of the 6th inst., when the name of the giver was not revealed, but it was stated that the money was given as an expression of gratitude for their Majesties' recent visit to the South Wales colliery district and to the King Edward VII.'s Hospital at Cardiff. It is now made clear by Colonel Bruce Vaughan's letter that the donor is Mr. W. James Thomas, J.P., of Brynawel, Ynyshir, already one of the most munificent benefactors of the King Edward VII.'s Hospital, Cardiff, and a gentleman who backs up his monetary gifts by a personal interest in the institution. It is greatly to be desired that Mr. Thomas's public-spirited action will be speedily followed by other wealthy men of the district, so that the estimated sum of £50,000 may be acquired to complete the entire scheme of buildings for the medical school. This would mean the provision of a complete medical school on the site of the old University Buildings in Newport Road, which are within a few hundred feet of the hospital, leaving the more distant site of the new buildings in Cathays Park free for the full development of the other branches of University work which are not dependent upon the co-operation of the hospital.

### SECRET REMEDIES FOR SPECIFIC DISORDERS.

Having heard evidence with regard to the proprietary medicines recommended for disease in general, the Select Committee on proprietary remedies is now engaged in the examination of witnesses who have made observations on specific forms of quackery. Dr. R. E. Crosse drew the attention of the Committee to the fact that there is a very large amount of advertising of appliances and preparations directly connected with sexual matters. He classified these as articles advertised for female irregularities, procuring abortion, preventing conception, nervous debility, and venereal diseases; but the Chairman decided that the evidence should be heard *in camera*. Mr. P. Macleod Yearsley, F.R.C.S., in his evidence, dealt with deafness "cures" advertised in the press. He said he had seen patients at hospitals who were absolutely incurable, but had been treated by various advertising quacks. He mentioned the case of a servant girl who had paid an "institute" twelve guineas in fees. New advertisements of deafness remedies were constantly appearing, and all the patterns of ear-drums advertised were bad.

### THE ADMINISTRATION OF FEVER HOSPITALS.

We direct the attention of those who are interested in the working and management of fever and isolation hospitals to the series of articles by Dr. Knyvett Gordon. The first article of the series appeared last week, and the second, dealing with isolation methods, appears on page 405 of this number. The series will be illustrated, and will treat the whole subject of the administration and working of isolation hospitals in a broad, practically suggestive manner, and should therefore prove of value to all hospital workers. Criticism of the points raised and of the various suggestions made is invited, and we shall be glad to publish communications dealing with the series if they are helpful and suggestive in the same manner as the original articles.

### "VISITORS" AT OPERATIONS.

The presence at operations of untrained persons generally was deprecated at the July meeting of the Board of Management of King Edward VII.'s Hospital, Cardiff, when the Chairman (Major-General Lee) proposed: "That the Medical Board be asked to draw up and submit for the approval of the Board of Management regulations governing the presence at operations of nurses and enrolled members of first-aid societies not on the staff of the hospital." General Lee said that it was well to have a definite understanding on this matter, as applications for educational facilities were frequently received from members of first-aid and similar societies. After some discussion it was contended that it was quite outside the province of first-aid members to witness operations or to enter operating theatres. Ultimately it was agreed to adopt the resolution, amended so as to limit such privileges to qualified medical men, medical students, and trained nurses. Can any responsible person be found to take a less stringent view?

### THE INSTITUTIONAL TRAINING OF THE FEEBLE-MINDED.

The report of the Superintendent of the Western Counties Mental Hospital, Starcross, is a very interesting document as showing with engaging simplicity what the institutional training of the feeble-minded really means to-day. Mr. Locke urges the importance of establishing custodial homes for unimprovables, whose presence, he says, among the new cases is inevitable from the too favourable reports of their relations who desire them to have institutional care. "We never willingly discharge," he adds, but the higher-grade cases as being less distinguishable from normal individuals generally deteriorate when launched into the world. It is for these that he recommends the establishment of colonies. The feature of the training is manual work, and the patients largely make each other's clothes, and supply the various needs of the institution, each child being usually taught two trades. The girls make Honiton lace, and nine medals were won by pupils at the Devon Arts and Crafts Exhibition last year. Recreation and amusement are lavishly organised; an aviary is kept and owned by the boys, while the orchestra is a recognised institution. Mr. Locke also, we are glad to note, refers to the recreation of the attendants, for whom, as can only be expected, Starcross cannot provide much amusement after working hours. This means that the institution must itself, in some sort, meet the need, which is greater probably among mental hospital attendants than among any other class of hospital worker, and a beginning has been made by a careful redecoration of their mess-room. As far as possible, however, it is, of course, essential that attendants should find relaxation in that changed atmosphere which only absence from the institution can afford fully. Dr. J. H. Iles, the medical officer, is a London Hospital man. The report is well illustrated, but we think it a mistake to group all the illustrations in the middle, and a full list of the staff does not appear to be comprised in it. Small changes, perhaps, but some which the secretary should see effected.

### A NEW TELEPHONE SERVICE AT CANE HILL.

It is proposed to instal at Cane Hill Mental Hospital a new internal telephone system, and the first part of the work is to be proceeded with at once at a cost of £125. The existing apparatus was installed over twenty years ago, and is now inadequate. There is no telephonic communication to the wards, and only the most important of the officers' quarters and offices and some only of the detached buildings are connected. Messages for the wards have to be taken by a messenger. The existing wiring is in open casings fixed to the corridor and gallery walls or carried under the floors, and damp, as readers of our recent article on short-circuits will vividly remember, occasions frequent leakages leading to an unsatisfactory service. It is proposed gradually to instal a telephone system similar to that in use in the more recent of the mental hospitals.

### A POPULAR APPOINTMENT.

In appointing Mr. H. K. Anderson, M.D., F.R.S., to the Mastership of Caius College, Cambridge, the electors made a choice which is not merely popular, but far-sighted and thoroughly admirable from every point of view. To all Cambridge medical students of the last twenty years Dr. Anderson is well known as a patient and assiduous teacher and a type of the modern don who does credit to his college, his university, and the medical profession. To physiologists his name stands for researches of the first order and a very high scientific reputation. And to Cambridge generally he is known for business acumen, perpetual energy in all that pertains to the welfare of Cambridge and Cambridge men, and a full measure of humanity. Dr. Anderson comes of a scientific stock, for he is related to the Haldane family, which produces physiologists as well as Lord Chancellors.

### THE SATURDAY FUND AND BOLINGBROKE HOSPITAL.

Sir Savile B. Crossley, Bart., presided at the quarterly meeting of the Hospital Saturday Fund. The Distribution Committee reported that on January 20 the Board authorised the withholding of the grant of £50 apportioned to the Bolingbroke Hospital until the Distribution Committee are satisfied with the experimental arrangements with the hospital then being tested. The Committee regretted that they were still dissatisfied with the manner in which patients recommended by the Fund were treated, and consequently recommended that the grant be cancelled and paid into the Distribution Committee's account for providing accommodation elsewhere. Mr. Davis asked whether the Bolingbroke Hospital had been notified of the dissatisfaction of the Fund as to the insistence of that institution upon the "letters" issued by the Fund being signed by a local medical practitioner before presentation at the hospital. He added that the Bolingbroke had articles of association which provided that neither out-patients nor in-patients should be admitted without a recommendation from a local medical man. Mr. W. F. Chalkley referred to the great and good work which had been done by the Bolingbroke Hospital, and expressed the hope that they would ultimately bring themselves into line with other institutions so as to receive awards from the Fund.

### STREET COLLECTIONS—A SUGGESTION.

Now that the result of the effort on "Alexandra Day" has been made public a correspondent sends us a suggestion for the consideration of all those who organise street collections on behalf of hospitals. Many a man, it is thought, does not now give to such collections because he is asked perhaps a dozen times; and as he does not like to give to one and to refuse others he therefore gives in many cases to none at all. If, however, collectors followed the idea of the promoters of Alexandra Day and gave a small badge or artificial flower it might be worn for the rest of the day as a "passport" to non-interference. We believe, however, that the Alexandra Day promoters have only extended a practice that in isolated cases has been used successfully before.

### A FORGOTTEN HEROINE.

What has been the result of the recent letter to the papers in which Mr. Percy White pleaded the cause and the necessities of Madame de Ronsard, a French lady who not only assisted Miss Nightingale in the Crimea, but was also on duty in the Prusso-Danish War and during the horrors of the Commune in Paris? In both of these latter outbreaks she was herself wounded whilst attending the wounded under fire. Madame de Ronsard received two medals from the French authorities, and also an *Etat de Service*, or official acknowledgment of her services. She has been three times married, and has been living for some years in Cairo with her third husband, M. Pater, an Italian taxidermist. Unfortunately, he and she fell ill together last year, and this double misfortune has brought them to the brink of financial ruin. Thus it happens that at the age of eighty-four this heroic woman is in actual and urgent need of help. It appears that her father, a Frenchman, was born in England in 1800 and married an English lady. Although he remained French, and his daughter therefore retained his nationality, her marriage to an Italian and her expatriation from France stand in the way of her obtaining a pension from the Republic. She has even less claim on the British Government, and so the only resort would appear to be private generosity. We cannot help thinking that an appeal to the generosity of the French public on behalf of their compatriot would also be productive of help and sympathy. Having largely helped to resuscitate the now very successful Nightingale Memorial Fund, we are glad to draw further attention to the work and need of her French colleague.

### DEATH OF A VETERAN ARMY DOCTOR.

The death is reported of the Hon. Charles George Drummond Mosse, for many years Deputy Surgeon-General of the Army Medical Department. He was eighty-two years of age, and had entered the Army in 1858 after graduating at Trinity College, Dublin, and served in the Gambia in 1866, where he was mentioned in despatches. Later on he took the diplomas of L.R.C.P.E. and F.R.C.S.I., and was made a Companion of the Order of the Bath. For twenty-five years he served as Superintending Medical Officer of the Colonial Medical Department, and was for some time a member of a Colonial Legislative Council. In 1897 he retired, and was gazetted C.M.G.

### THE AFTER-CARE OF MENTAL PATIENTS.

The objects of the After-Care Association are to assist recovered cases on their discharge from mental hospitals throughout the country. At two recent meetings—the first at Highgate in the gardens of All Saints' Vicarage, by the kind invitation of the Rev. and Mrs. F. H. A. Hawkins, was addressed by Sir George Savage, M.D. (treasurer), Miss Helen Webb, M.B., and Mr. H. Thornhill Roxby; the second at Collingwood Towers, Camberley, by the kindness of Colonel and Mrs. Harris, was addressed on the work of the Association by Sir James Crichton-Browne, F.R.S. (Lord Chancellor's Visitor in Lunacy); Dr. Nicolson, C.B., and the Hon. John Mansfield, Lord Chancellor's Visitors in Lunacy, Colonel Harris, R.A., and Mr. H. Thornhill Roxby also spoke. Branches of the Association were formed in both places. With the problem of the mentally afflicted now more pressing than ever, with our own plea for research work to utilise the present almost complete waste of clinical material in our mental hospitals, we may well note the efforts of this society, which if it but touches one fringe of the subject, yet is attempting to obviate some of the incidence of recurrent insanity.

### PROMOTIONS AT GUY'S HOSPITAL.

At the recent General Court of the Governing Body of Guy's Hospital, Mr. Charters Symonds, M.D., M.S., F.R.C.S., was elected consulting surgeon to the hospital on his retirement from the position of senior surgeon. Mr. Symonds will take with him the good will and good wishes of several generations of Guy's men, to whom he has always been a lofty example, a valued friend, and a fine teacher. To fill the vacancy thus left, Mr. F. J. Steward, M.D., F.R.C.S., senior assistant surgeon, is promoted to the full staff. Mr. Steward's name is known far beyond the walls of Guy's, and he has well earned the promotion which he now receives. In his place on the assistant staff, Mr. R. Davies-Colley, M.C., F.R.C.S., is elected fifth assistant surgeon. Mr. Davies-Colley bears a name which ensures him a welcome at Guy's and indeed anywhere in surgical circles in London; and his record leaves no room for doubt that he will maintain its traditions most worthily.

### CHANGES AT THE "DREADNOUGHT" AND OTHER HOSPITALS.

On the resignation of Sir William H. Bennett of his post as senior surgeon to the "Dreadnought" Hospital he has been appointed consulting surgeon to the institution, an honourable distinction which he enjoys also at St. George's. To the vacancy thus created Mr. C. C. Choyce succeeds by promotion from the assistant staff. Mr. Choyce is the well-known Dean of the Post-Graduate School attached to the hospital. His place as assistant surgeon is filled by the appointment of Mr. P. P. Cole, F.R.C.S., an old Guy's man, and at present surgical registrar to the Cancer Hospital and surgeon to the West Ham Hospital. At Westminster Hospital Dr. D. H. de Souza becomes assistant physician: this is not in succession to the late Dr. Murrell, whose place has not yet been filled. Dr. de Souza is an old University College Hospital man, who has been medical registrar for some time at Westminster. At the West London Hospital Dr. Harold Pritchard has succeeded Dr. Seymour Taylor on the full staff, and Dr. S. A. Owen has been elected assistant physician in his place. He is a Cambridge and University College Hospital man, and is already out-patient physician to the Victoria Park Hospital, E.

### AN ASSISTANT SECRETARY'S AUTHORSHIP.

Captain Eustace Lees, assistant secretary of the Wolverhampton and Midland Counties Eye Infirmary, has just published through Simpkin, Marshall and Co. a pamphlet on "The Preservation of the Teeth," price 1s. Two medical friends are stated to have assisted him on the technics of the question, but the author's aim is rather to explain a method of recording the treatment which a patient's teeth have experienced than to deal with dental therapeutics. The pamphlet therefore contains illustrated diagrams, enabling each tooth to preserve its own medical history.

### THE CONVERSION OF CLARE HALL HOSPITAL.

By the opening on the 15th inst. of the Clare Hall Hospital, South Mimms, Barnet, as a sanatorium for the treatment of consumptives in the incipient stage, the County of Middlesex has increased its accommodation for dealing with pulmonary tuberculosis. The hospital was, in 1903, acquired under provisional order by the Middlesex District Councils Association, a union of some twelve constituent authorities, for the treatment of smallpox cases, but subsequently complaints were made of the heavy expenses of the upkeep of the institution and of the stand-by staff, which was necessary in case of emergency, and a movement was started to reduce the high cost of maintenance and to increase the utility of the institution. As a result of representations made, the Local Government Board issued their provisional order permitting the adaptation of the hospital for the reception and treatment of consumptives in the incipient stage, and, this course having been adopted, the hospital was accordingly open for the reception of such consumptives on the 15th inst. As an experiment, twenty-four patients—twelve of each sex—are to be admitted for treatment.

### PROGRESS OF THE LAMBETH INFIRMARY.

A most interesting return showing the general work performed at one of the great Metropolitan infirmaries, prepared by Dr. Baily of the Lambeth Infirmary, has been presented to the Guardians. The total number of admissions in one year numbered 4,193, and of these, 716 patients died, 2,297 were discharged relieved, 589 discharged unrelieved, and 591 remained in the institution. There were 148 patients admitted suffering from injuries and ten from the effects of poisoning. The operations performed amounted to 274. The Chairman, Mr. Frank Briant, L.C.C., said that these figures proved conclusively that the infirmary was doing identically the same work as a general hospital. He suggested that copies of the return should be circulated amongst those interested in the National Insurance Act, for the possible effect that Act might have upon infirmaries and hospitals. Mr. Briant is right to be proud of the amount of work which the Lambeth Infirmary is doing, but why did he not go further and follow up his suggestion of circularising the public by giving his opinion as to the effects which the new-born Act is likely to have upon the infirmaries generally? They have been so far without a spokesman, and, though they are publicly supported institutions, the problems which they anticipate might well be stated. Why not by Dr. Baily or Mr. Briant?

### HOSPITAL PETS.

Most hospitals possess one or more animals which may be regarded as institutional pets. The ward cat is an indispensable member of every hospital staff, and there are generally other less useful animal pensioners attached to our great hospitals. One provincial institution owns and makes much of a tortoise that has a history. Now the Royal Hospital for Incurables, Putney Heath, will have equally interesting pets. When haymaking operations were in progress in the meadow belonging to the hospital, a man with a scythe unwittingly guillotined a hen pheasant, which was sitting on her nest in the long grass. In the nest were found ten young pheasants, which the head gardener is doing his utmost to rear.

### PUBLIC PHARMACISTS ON THEIR DEFENCE.

Pharmacists in public institutions are not slackening in their interest in the Insurance Act. At a recent meeting in the premises of the Pharmaceutical Society, when the President of the

Chemists' Friendly Society explained how that society hoped to administer the benefits in the most advantageous manner, Mr. J. H. France (St. George's Dispensary) and Mr. F. E. Bullen (Brixton Dispensary) were amongst the speakers. A committee of six was appointed to assist the society in the London area, and three of these are Metropolitan Poor-Law dispensers, Miss C. A. Andrews (Paddington District Dispensary), Mr. G. W. Gibson (St. Pancras South Infirmary), and Mr. R. W. Lindsey, F.C.S. (District Dispensary, St. Mary's, Islington), the Chairman of the Public Pharmacists' Association. We also learn that the Public Pharmacists' Association, having failed to obtain representation on the Pharmaceutical Standing Committee on Insurance, has elected a special committee to deal with insurance points affecting specially their calling. This committee, it is stated, will be able to communicate direct with the Commissioners, and will seek to have the position of pharmacists clearly defined should there be institutions established under the Act.

### A COMMITTEEMAN'S RESIGNATION AT WORCESTER.

Mr. C. Raymond has tendered his resignation of his place on the Finance Committee of the Worcester General Infirmary, giving as his reasons that at the last meeting of the Executive Committee £350 was voted for *x*-ray apparatus and £135 for electric lighting, in spite of the fact that Dr. Crowe had said the latter was "not absolutely necessary." The maintenance and increased cost of those two would be at least £80 per annum. The annual expenditure of the institution was now over £10,000, and, at the present rate, he said, all their remaining saleable stock (£20,000 Corporation stock) would be gone in three years, and two or more wards closed for want of funds. His repeated financial advice was rejected, and he therefore declined to be a party to the wrecking of that worthy institution. His resignation was accepted. Further discussion followed on the financial position of the infirmary, Alderman Leicester regretting that no announcement had been made to remove the wrong impression entertained by the public as to the real state of the finances. Reference was made to an inquiry instituted by the Bishop which was to have resulted in a letter to the press. The inquiry was set on foot in consequence of a misconception having arisen that the Committee sold ear-marked legacies. The Bishop had also intended to make an appeal to the general public, but considered the moment inopportune, and the matter was postponed. One speaker said that it might be assumed that the Bishop was satisfied that legacies were not being improperly dealt with. It was proposed that the appeal should be made now, as there must be many persons in the country able to help them. Alderman Leicester laid great stress on the necessity for removing all misconceptions from the minds of the public as to the state of their finances. They ought to tell the public, he said, that they had undertaken more charitable work than could be performed with the funds available at the present time.

### A PHYSIOLOGIST'S PENSION FROM THE CIVIL LIST.

Among the list of pensions granted during the year ended March 31, 1912, and payable under the provisions of section 9 (1) of the Civil List Act, 1910, which has been issued as a Parliamentary Paper [167], we note the name of Dr. George James Burch, £120, "in recognition of the value of his researches in physics and physiology, and in consideration of his inability to continue his professional work owing to ill-health."

### NEW APPOINTMENTS AT THE COLONIAL OFFICE.

Sir Patrick Manson has resigned his position as senior medical adviser to the Colonial Office and Crown Agents for the Colonies, and has been succeeded by Sir John Rose Bradford, M.D., F.R.C.P., who is physician to the Seamen's Hospital ("Dreadnought") and to the University College Hospital. Sir John was gazetted a K.C.M.G. last year, and is specially known for his work in connection with the kidneys, and not for his researches in tropical medicine. Dr. Charles W. Daniels, who is a well-known lecturer at the "Dreadnought" School of Tropical Medicine, and who was formerly in the Fiji and British Guiana Medical Services, a member of the Royal Society's Special Commission on Malaria, and late director of the Kualo Lumpur Institute for Medical Research, becomes assistant medical adviser to the Colonial Office. Dr. Daniels' teaching is familiar to very many post-graduate students, both medical men and officials in the Colonial Service, who will join with us in congratulating him on his well-earned promotion.

### THIS WEEK'S DRUG MARKET.

No improvement is noticeable in the Drug and Chemical markets, and little or none is expected until the holiday season is over. The labour trouble at the docks is not now causing so much inconvenience in the Drug market, and less difficulty is experienced in obtaining delivery of goods. Of changes in value there have not been many of importance. The hot weather has naturally increased the demand for seasonable drugs, but not sufficiently to account for any marked advances in prices. Citric acid and tartaric acid are in good request, as also is oil of lemon, which seems likely to become dearer. Cod-liver oil is not in demand at present; the general opinion is that the price of this oil has reached its lowest level. The position of opium is unchanged, buyers still holding off in the hope that prices will be much reduced. Oil of aniseed has a downward tendency in value. Chamomiles are rather dearer, but the new crop is expected shortly. Saffron is higher in price. Still higher prices are expected for eucalyptus oil. Linseed oil is much lower in value.

### TO OUR READERS.

Contributions are specially invited from any of our readers to these columns. They should deal with topical subjects and news. They must be authenticated for the information of the Editor only. The minimum payment if published is 5s. There is no hard-and-fast rule as to space, but notes of about twenty lines in length are preferred.

